# Post-sepsis syndrome – an evolving entity that afflicts survivors of sepsis

**DOI:** 10.1186/s10020-019-0132-z

**Published:** 2019-12-31

**Authors:** Zachary Mostel, Abraham Perl, Matthew Marck, Syed F. Mehdi, Barbara Lowell, Sagar Bathija, Ramchandani Santosh, Valentin A. Pavlov, Sangeeta S. Chavan, Jesse Roth

**Affiliations:** 1 Laboratory of Diabetes and Diabetes-Related Research, Feinstein Institute for Medical Research, Northwell Health, Manhasset, NY USA; 2 Sackler School of Medicine, Tel Aviv University, Tel Aviv, Israel; 3 Center for Bioelectronic Medicine and Biomedical Science, Feinstein Institute for Medical Research, Northwell Health, Manhasset, NY USA; 4 Donald and Barbara Zucker School of Medicine at Hofstra/Northwell, Manhasset, NY USA

**Keywords:** Post-sepsis syndrome, Sepsis survivors, Sepsis sequelae, HMGB1

## Abstract

**Background:**

The sequelae of sepsis were once thought to be independent of sepsis itself and assumed to be either comorbid to sick patients or complications of critical illness. Recent studies have reported consistent patterns of functional disabilities in sepsis survivors that can last from months to years after symptoms of active sepsis had resolved.

**Body:**

Post-sepsis syndrome is an emerging pathological entity that has garnered significant interest amongst clinicians and researchers over the last two decades. It is marked by a significantly increased risk of death and a poor health-related quality of life associated with a constellation of long-term effects that persist following the patient’s bout with sepsis. These include neurocognitive impairment, functional disability, psychological deficits, and worsening medical conditions.

**Conclusion:**

This “post-sepsis syndrome” has been the subject of active preclinical and clinical research providing new mechanistic insights and approaches linked to survivor well-being. Here we review important aspects of these research efforts and goals of care for patients who survive sepsis.

## Background

Post-sepsis syndrome is a relatively newly described pathologic entity. The earliest descriptions of an ensuing syndrome, recorded at the turn of the twenty-first century, described consistent cognitive, psychological, physical, and medical defects following severe sepsis. The sequelae had first been thought to be independent of the sepsis; patients predisposed to the development of sepsis were also predisposed to cognitive and functional impairment. Sepsis as a disease-related entity, once thought to be due to the dissemination of pathogens, has since been redefined as dysregulation of the host immunological response. Similarly, the sequelae of sepsis need to be redefined as their own pathological entity. Sepsis does not end with resolution of symptoms – there are long term sequelae often with devastating consequences. In this review we describe important aspects of the post-sepsis syndrome and its pathophysiology, provide an overview of the effects of sepsis on long-term patient function, and outline considerations for therapeutic strategies. References for this review were identified and selected through searches of PubMed for the search term “post-sepsis syndrome.” Articles with relevant titles and abstracts were analyzed and their reference lists were also searched for relevant publications.

## Introduction

### History of Sepsis

Sepsis was originally characterized as organ failure that develops as a result of dysregulated host responses to infection (Singer et al. 2016). The concept of sepsis has since evolved. Until the second half of the twentieth century, the pathogenesis of sepsis was thought to be due to the uncontrolled dissemination of pathogens. In 1896, Pfeifer discovered “endotoxin,” a heat-stable component of the bacterial cell wall, that he held responsible for the toxic effects of gram-negative microbes. Endotoxin was soon linked to the ability of several bacterial species to cause fever. By 1926, descriptions of microthrombi in endotoxic shock led to an association between infection and coagulopathy; this phenomenon was later to be termed disseminated intravascular coagulation. There still remained a missing mechanistic explanation as to how infection activates the coagulation cascade. The proposed solution to this pathophysiological query was the discovery of cytokines in the 1970s and 1980s. Cytokines are soluble factors generated by a broad range of host cell types that mediate immune function and other biological activities. These factors modulate the immune response during sepsis. Interleukin-1, discovered in 1971 (Gery et al. 1971), and tumor necrosis factor, discovered in 1975 (Carswell et al. 1975), were among the earliest cytokines identified; they were then found to play central roles in sepsis pathophysiology. Nawroth was one of the first to demonstrate that interleukin-1 could induce an endothelial procoagulant later known as tissue factor (Nawroth et al. 1986). In the decades that followed, the coagulation and inflammatory cascades were further interconnected by the rapid discovery of many other cytokines (Funk et al. 2009).

### Evolving definitions of sepsis

The pathogen-driven theory of sepsis was challenged, as it was found that as many as 40% of sepsis cases followed sterile tissue injury caused by non-infectious sources such as pancreatitis, severe trauma, burns, ischemia-reperfusion injury, and cancer (Vincent et al. 2013). In the early 1980s, the focus shifted toward the failure of the immune system and the resultant disequilibrium between immunosuppression and immunostimulation (Tracey et al. 1986). Resolution of the inflammation is necessary for return to immunological homeostasis and the well-being of survivors of sepsis. The biological response after sepsis involves tissue repair, rebalancing metabolic setpoints, reestablishing organ function, and generating mediators of a lasting adaptive immune response (Lewis et al. 2016).

Sepsis survival does not necessarily equate with restored quality of life or with improved outcomes for survivors. Recently, it has been established that patients have increased mortality and decreased quality of life even 2 years after the 28 day in-hospital mortality endpoint (Winters et al. 2010). This standard endpoint of mortality is problematic, as it fails to account for the long-term effects of immune and metabolic derangements, which are importantly linked to the increased mortality and decreased quality of life of sepsis survivors. Current literature supports a litany of continuing issues including neurocognitive, psychological, physical, and medical effects (Prescott and Angus 2018). A major goal is to gain a comprehensive understanding of sepsis sequelae and to identify new therapeutic approaches for survivors.

### The costs of sepsis

**Fig. 1:**
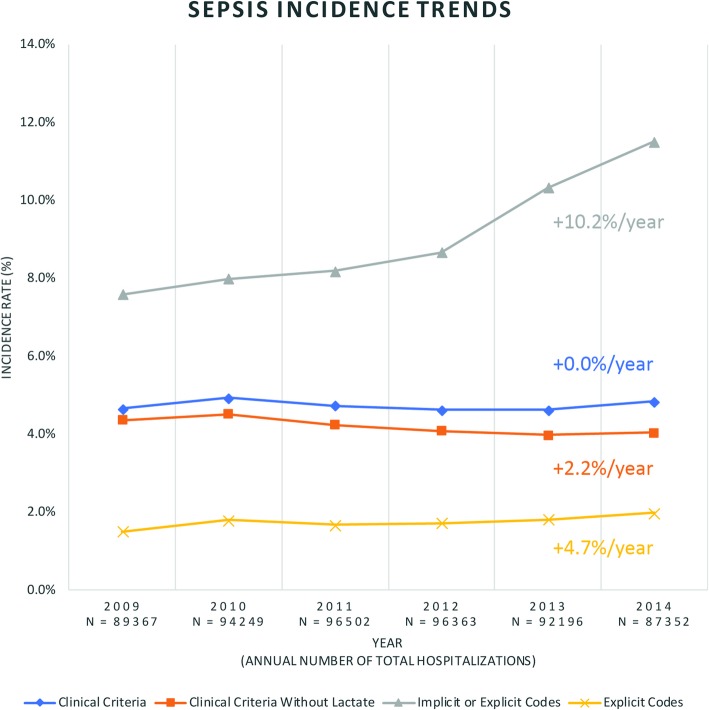
Sepsis incidence trends. Figure adapted with supplementary data and used with permission by authors (Rhee et al. 2017)

**Fig. 2:**
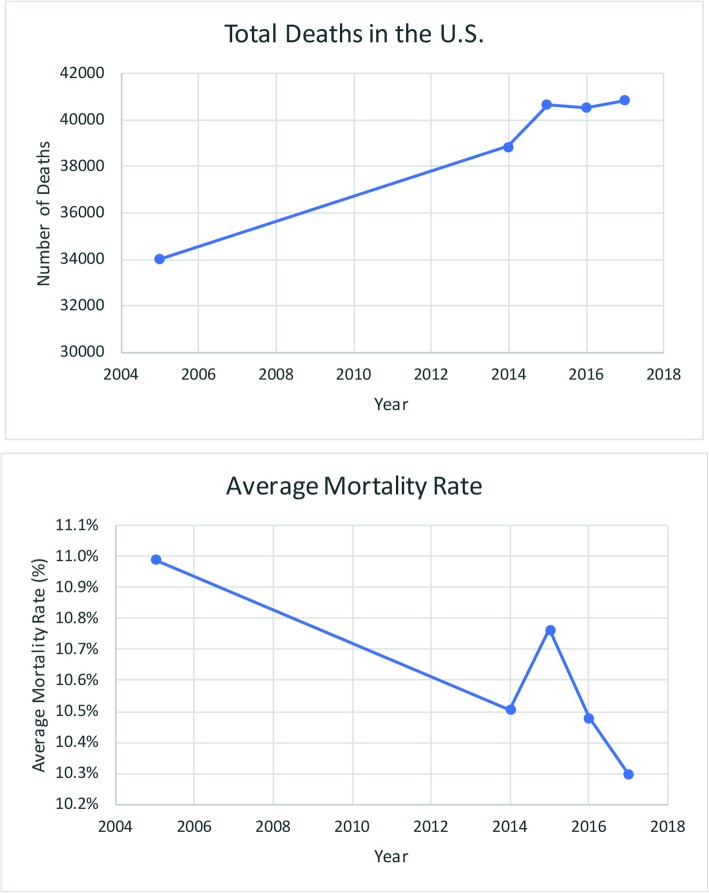
Septicemia mortality in the United States 2004–2017. **a** Total Deaths in the U.S. **b** Average Mortality Rate. Original figure based on data from the Center for Disease Control and Prevention. Septicemia Mortality by State: 2004–2017. National Center for Health Statistics 2017 January 11, 2019; Available from:https://www.cdc.gov/nchs/pressroom/sosmap/septicemia_mortality/septicemia.htm

**Fig. 3:**
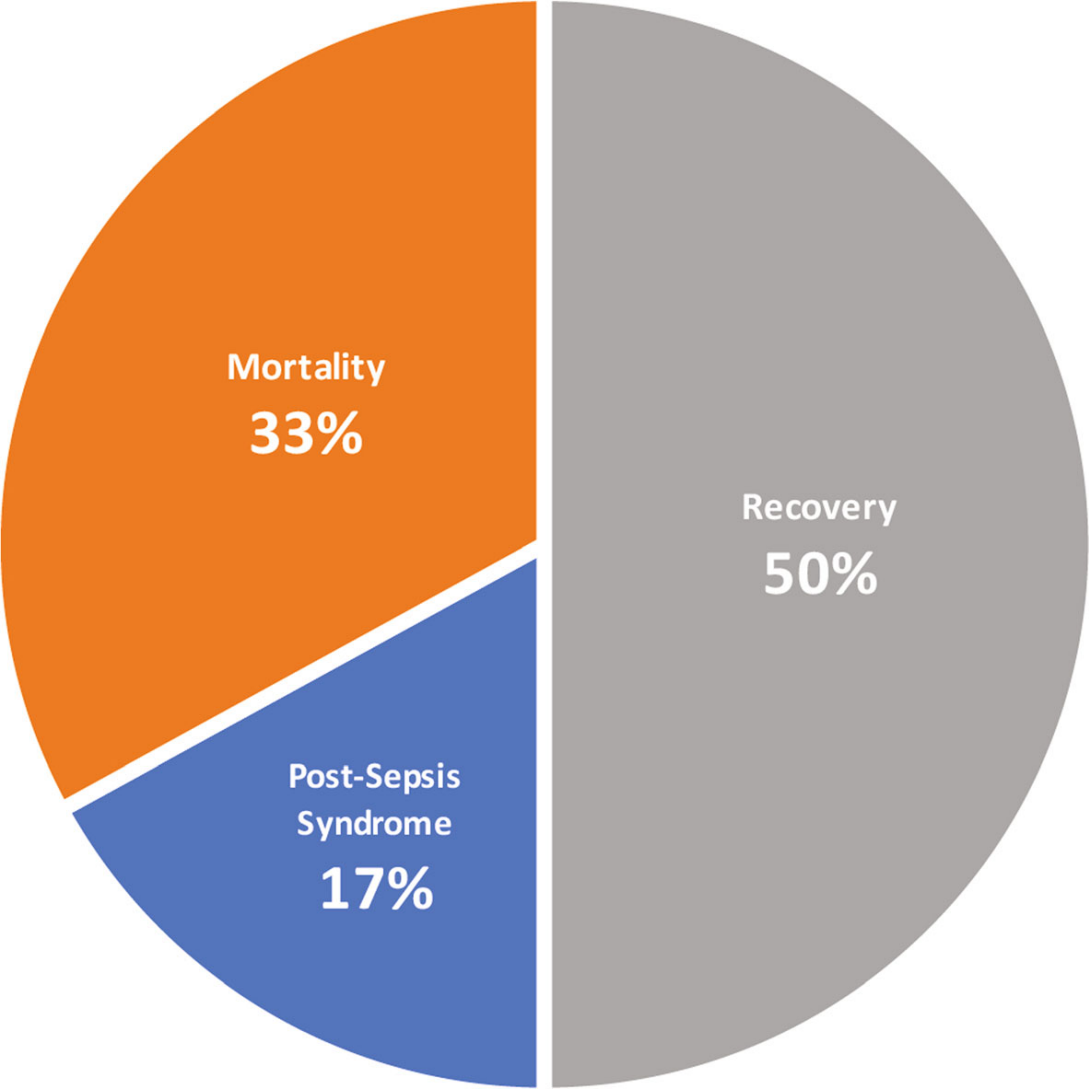
Patient outcomes following sepsis. Original figure. Approximately half of patients who survived a hospitalization for sepsis achieved a complete or near complete recovery at 2 years after discharge; one third of total patients died during this period; and one sixth of these patients remained with one or more of the serious, lasting complications of post-sepsis syndrome

There has been a substantial increase in the annual reported incidence of sepsis over the last two decades that has become a major challenge for physicians (Fig. 1) (Rhee et al. 2017). The reasons for the rise in the incidence of sepsis will be discussed below. Sepsis affects approximately 100 people per 100,000 and is diagnosed in 6 % of hospitalizations (Rhee et al. 2017; Moss 2005). Along with rising numbers, increased survivorship has warranted examination of life after sepsis (Fig. 2) (Fleischmann et al. 2016). Every year in the United States, 500,000 new cases join a pool of over 2.5 million survivors of sepsis (Iwashyna et al. 2010). According to a recent report, approximately half of patients who survived a hospitalization for sepsis achieved a complete or near complete recovery at 2 years after discharge; one third of these patients died during this period; and one sixth of these patients remained with one or more of the serious, lasting complications described above (Fig. 3) (Prescott and Angus 2018). Note that in those patients who are able to regain function, often the recovery is not complete.

The management of sepsis has been considered the costliest reason for admission to hospitals in the United States, with rising individual cost proportionate to severity. In 2013, sepsis accounted for more than $24 billion in U.S. hospital expenses, or 13% of total U.S. hospital costs (Paoli et al. 2018). Survivors with residual physical disabilities yield a vast increase in cost of healthcare over the years that follow (Fried et al. 2001). Sepsis has become a costly burden on the health-care system due to its rising number of cases, repeated treatment failures, and debilitating long-term sequelae for survivors.

Until now, these sequelae have been poorly categorized due to two primary challenges. The first is defining an endpoint (or endpoints) that takes into account mortality and lasting sequelae that are directly caused by sepsis. Establishing additional time points would allow for better categorization of mortality and long-term sequelae as processes that continue beyond the initial insult. The second challenge is to determine which sequelae ought to be included as arising from the insult itself – acute sepsis. This is not a simple task, as sepsis patients have many comorbidities and previous hospitalizations that confound whether or not certain sequelae are caused by sepsis itself or by extraneous factors. Here we circumvent this issue by only describing sequelae that have been well-categorized in the current literature as arising from sepsis. The sequelae of sepsis outlined below will be referred to as the post-sepsis syndrome, a distinct entity with well-defined consequences. We hope that doing so will help guide potential treatments for this highly prevalent constellation of symptoms secondary to sepsis.

### Neurocognitive derangements

#### Inflammatory mediators and the immune response to sepsis

Experts estimate that 25 to 50% of survivors of severe sepsis show considerable cognitive impairment (Annane and Sharshar 2015; Chavan et al. 2012). Problems with memory, learning, concentrating, and decision-making affect the daily lives of patients, their caregivers, and their families. The neurological sequelae in sepsis survivors are not fully understood. The proposed mechanisms of neurological deterioration and other disability in survivors involve a complex interplay between immune and metabolic alterations. Ongoing pre-clinical and clinical research has identified a major role for dysregulated host immune responses. High mobility group box-1 (HMGB1), a cytokine central to sepsis pathophysiology, has been identified as an important mediator of post-sepsis neurological manifestations (Chavan et al. 2012). HMGB1 was first discovered as a nuclear protein that regulates transcription factors in the cell nucleus. Recently, it has also been recognized as an extracellular messenger akin to cytokines and a *late* mediator of the inflammatory cascade. HMGB1 released by macrophages interacts with toll-like receptors on neutrophils, thereby upregulating nuclear factor kappa-light-chain-enhancer of activated B cells (NF-kappaB) that stimulates further synthesis and release of cytokines (Klune et al. 2008). In addition, the action of HMGB1 on toll-like receptors stimulates nicotinamide adenine dinucleotide phosphate oxidase (NADPH oxidase) to produce reactive oxygen species. In infections, these processes enhance destruction of engulfed bacteria by phagocytes (Park et al. 2006).

In contrast to early onset inflammatory mediators of sepsis (namely, tumor necrosis factor and interleukin-1), which return to baseline early in murine models, HMGB1 levels remain elevated for at least 4 weeks after experimental sepsis induced by cecal ligation and puncture (Chavan et al. 2012). Table 1 is a review of the major inflammatory mediators of sepsis and their roles. Brain pathology in mice revealed a reduction in the density of dendritic processes of hippocampal neurons in sepsis survivors as compared with controls. This atypical pattern was not present until after 2 weeks and continued for at least 4 months. Notably, the dendritic degeneration was not caused by acute sepsis but rather was a progressive phenomenon in survivors of severe sepsis. Dendritic processes play an integral role in synaptic plasticity and in memory; one proposed mechanism of cognitive impairment post-sepsis is the loss of hippocampal spine density. Administration of neutralizing anti-HMGB1 antibody conferred significant protection against the loss of dendritic spines. Memory impairment and brain pathology were both improved upon administration of anti-HMGB1 antibody to mice survivors beginning 1 week after onset of sepsis. Thus, there may be a window of opportunity following sepsis during which administration of anti-HMGB1 antibodies or other HMGB1 nullifying agents may prevent or even reverse neural impairment (Chavan et al. 2012).

**Table 1:** Inflammatory mediators of sepsis

Inflammatory mediators of sepsis	
High mobility group box-1 (HMGB1) – a late mediator of the inflammatory cascade; proposed driver of neurocognitive impairment after sepsis involves high serum levels; potential target for prevention of post-sepsis syndrome (Chavan et al. 2012) Interleukin-1 – an early mediator of sepsis; signals for the chemotaxis of leukocytes to sites of infection; causes a rise in body temperature (fever) via the thermoregulatory center in the hypothalamus (Faix 2013) Tumor necrosis factor-α – an early mediator of sepsis; signals for the chemotaxis of leukocytes to sites of infection; causes cachexia in malignancy and maintains granulomas in tuberculosis (Faix 2013) Interleukin-6 – a cytokine that causes fever and stimulates production of acute phase reactants (i.e. C-reactive protein, ferritin, fibrinogen, hepcidin) (Faix 2013) Interleukin-12 – a cytokine that induces the differentiation of T-cells and activates natural killer cells (Faix 2013) C-reactive protein – an acute phase reactant that produces complement fixation and facilitates phagocytosis; laboratory measurement used to monitor ongoing non-specific inflammation (Faix 2013)	

#### A landmark study of cognitive impairment after sepsis

Iwashyna et al. (2010) were early investigators who suspected that severe sepsis was responsible for a decline in cognitive function and sought to quantify the degree of impairment (Iwashyna et al. 2010). Detailed data on physical and cognitive function drawn from the Health and Retirement Study, a nine-year national survey of older Americans, was assessed both before and after an episode of sepsis. The results controlled for both the functional status of the patient before sepsis but also for functional trajectory. It was found that severe sepsis was associated with a 10.6 percentage point increase in the prevalence of moderate to severe cognitive impairment among survivors. Hospitalizations for reasons other than sepsis were associated with no change in moderate to severe cognitive impairment and were associated with fewer new limitations than those limitations seen in sepsis survivors. The worsening cognitive function in sepsis survivors was found to continue for at least 8 years. A review of published dementia (Ziegler-Graham et al. 2008) and sepsis (Angus et al. 2001) incidence rates for patients aged 65 years or older in the United States, suggests that nearly 20,000 new cases per year of moderate to severe cognitive impairment in the elderly may be attributable to sepsis. This vanguard study of a nationally representative cohort showed that severe sepsis is independently associated with lasting cognitive impairment.

#### Mechanisms of cognitive decline

The mechanisms behind the described neurological sequelae following sepsis involve a combination of cerebrovascular injury, metabolic derangements, and neuroinflammation. The acute phase of sepsis is marked by frequent abrupt variations in blood pressure that result in hemorrhage and ischemia within the brain parenchyma. Post-mortem studies have shown septic shock to be associated with ischemic neurons and hemorrhagic lesions in regions of the brain susceptible to hypoxia, such as the hippocampus (Sharshar et al. 2004; Sharshar and Gray 2003).

Dysregulation of metabolism, mainly uremia and hyperglycemia, are thought to also be major contributors to the cognitive impairment in sepsis. Guanidine compounds, such as creatinine, stimulate N-methyl-D-aspartate (NMDA) receptors and inhibit gamma-aminobutyric acid (GABA) receptors (De Deyn et al. 2009). The net excitotoxicity stimuli in the brain contribute to multiple brain pathologies including epilepsy, Alzheimer disease, Parkinson disease, and amyotrophic lateral sclerosis (Michels et al. 2015). High levels of glucose in the brain may play a role in cell apoptosis (Polito et al. 2011), activation of matrix metalloproteinases (Kamada et al. 2007), and disruption of the blood-brain barrier. Matrix metalloproteinases cleave collagen in the choroid plexus causing increased vascular permeability of the blood-brain barrier (Vandenbroucke et al. 2012). The magnitude of cognitive decline in survivors of acute respiratory distress syndrome 1 year after discharge from an intensive care unit was found to be proportional to the degree of stress-induced hyperglycemia noted during their stay in the intensive care unit (Duning et al. 2010; Hopkins et al. 2010).

The hallmark of neuroinflammation is activation of microglia and astroglia which produce cytokines (such as interleukin-6, interleukin-12, and tumor necrosis factor), reactive oxygen species, and dysregulated release of glutamate (Loane and Byrnes 2010). The sustained neuroinflammation during sepsis is associated with compromised integrity of the blood-brain barrier and accumulation of peripheral inflammatory mediators; these mediators remain elevated in survivors and substantially contribute to and exacerbate neuroinflammation (Yende et al. 2008). A large cohort study of patients with 10 years of follow-up found an association between increased levels of circulating inflammatory mediators (i.e. interleukin-6 and C-reactive protein) and cognitive impairment (Singh-Manoux et al. 2014). In a murine model of sepsis, increased levels of systemic pro-inflammatory markers (including tumor necrosis factor, interleukin-6, interleukin-1b, and HMGB1) and altered levels of metabolic molecules (including insulin, leptin, and plasminogen activator inhibitor-1) were related to persistent neuroinflammation in mice surviving sepsis (Zaghloul et al. 2017). Intriguingly, these peripheral and brain immunometabolic alterations coexist with impaired forebrain cholinergic signaling in mice that survive sepsis (Zaghloul et al. 2017). Brain cholinergic signaling has a well-documented role in the regulation of cognition and has also been shown to regulate peripheral inflammation and neuroinflammation (Chang et al. 2019; Lehner et al. 2019; Pavlov et al. 2018; Pavlov and Tracey 2017). These findings suggest that pharmacological and bioelectronic enhancement of brain cholinergic signaling may be therapeutically exploited for alleviation of inflammatory and cognitive derangements in survivors of sepsis (Zaghloul et al. 2017; Pavlov et al. 2018).

**Fig. 4:**
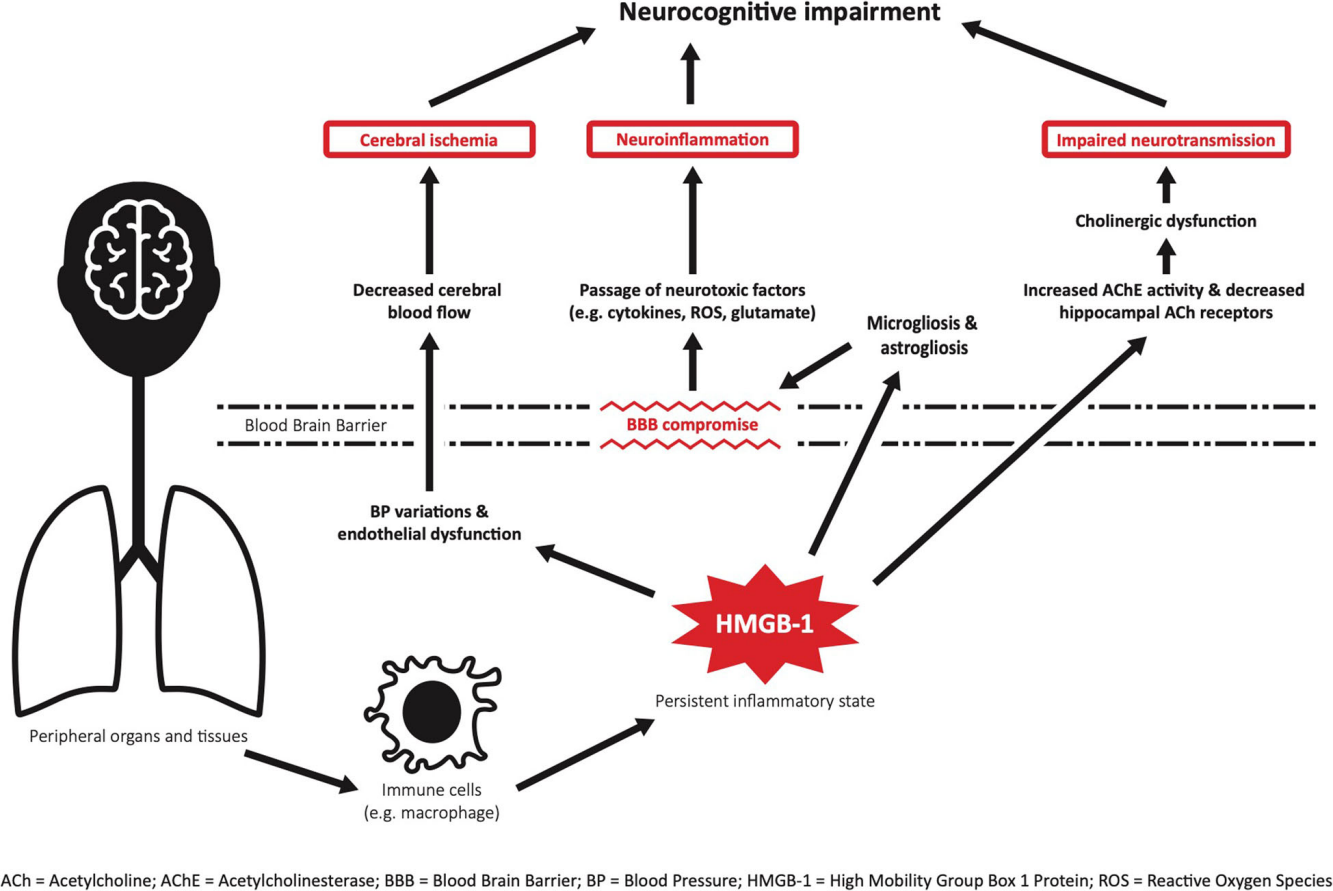
Mechanisms of cognitive decline after sepsis. The neurocognitive effects of sepsis are caused by a combination of cerebral ischemia, neuronal dysfunction, and neuroinflammation. These three contributory pathways are associated with persistently elevated levels of HMGB1, a cytokine secreted by immune cells (e.g. monocytes, macrophages, and dendritic cells) during the late stages of sepsis. Systemic endothelial dysfunction and variations in blood pressure lead to poor blood flow to the brain and contribute to cerebral ischemia. Cholinergic dysfunction is related to increased acetylcholinesterase activity and a decrease in receptor density in the hippocampus; this pathophysiology is a major contributor to impaired neurotransmission. Activation of microglia and astroglia produce increased inflammatory mediators (tumor necrosis factor, interleukin-6, and interleukin-12) and is associated with compromised blood-brain barrier integrity that allows for the passage of neurotoxic factors (cytokines, reactive oxygen species, and glutamate) and sustained neuroinflammation

At present, there are two hypotheses at play for the mechanism of long-term cognitive decline post-sepsis: (i) the vascular hypothesis posits that multiple cerebrovascular accidents play a dominant role; (ii) the neurodegenerative hypothesis maintains that microglial activation, combined with blood-brain barrier impairment, lead to sustained chemotaxis of neurotoxic mediators that impair neurotransmission (Fig. 4) (Annane and Sharshar 2015).

### Neuropsychiatric consequences

#### Emotional issues after sepsis

Some studies have found that surviving sepsis is associated with lasting effects on one’s mental health (Davydow et al. 2013; Gawlytta et al. 2017). The cerebrovascular injury and neuroinflammation that follow sepsis may be the responsible processes, explaining the psychological sequelae of sepsis and how they similarly affect both sexes. There are few studies that specifically examine psychiatric issues after sepsis and further investigation is necessary in this area of post-sepsis syndrome research. However, there have been several studies that describe the neuropsychiatric sequelae that belong to an entity called post-intensive care unit syndrome. Sepsis accounts for a large segment of intensive care unit survivors and are thereby often included in the entity of post-intensive care unit syndrome. In brief, post-intensive care unit syndrome is a constellation of issues that is often seen in survivors of critical illness; these include cognitive impairment, neuromuscular weakness, cachexia, chronic pain, dysphagia, and psychiatric disorders (Rosendahl et al. 2013). We do not purport that the two syndromes are one and the same, but there exists significant overlap among patient populations in each category. For the purposes of this paper, the remainder of this section will discuss the neuropsychiatric consequences of surviving critical illness.

Patients have been found to suffer ongoing psychological issues after a bout of critical illness (Rattray et al. 2005). These negative emotional outcomes include anxiety, depression, and post-traumatic stress disorder. A lack of recall for events during the illness and uncertainty about future events may predispose such patients to anxiety and depression (Jones et al. 1998). Anxiety has been reported by up to 43% of patients and depression by up to 30% of patients (Eddleston et al. 2000; Scragg et al. 2001). Three recent metanalyses found anxiety in 32% of survivors within two to 3 months (Nikayin et al. 2016), depression in 29% of survivors within two to 3 months (Rabiee et al. 2016), and post-traumatic stress disorder in 44% of survivors within one to 6 months (Parker et al. 2015).

Depressive features often exacerbate worsening functionality or limit rehabilitation (DiPietro 2001). Both severe sepsis and depression are predictors of cognitive and functional decline in survivors (Spitzer et al. 1995; Steffens et al. 2006). Post-traumatic stress reactions have also been recognized as occurring in response to critical illness (Mayou and Smith 1997). Events that induce post-traumatic stress disorder are viewed by survivors as threatening to life, out of their control (Foa et al. 1989), and atypical to the human experience (Brewin et al. 1996). Between 14 and 27% of intensive care patients may develop a post-traumatic stress reaction (Parker et al. 2015; Cuthbertson et al. 2004; Schelling et al. 1998) that may persist for several years (Kapfhammer et al. 2004). Importantly, illness severity, as measured by the Acute Physiology and Chronic Health Evaluation (APACHE II) scoring system, was not associated with psychological outcomes. While many survivors had preexisting conditions, a Danish study of 9912 critically ill patients found that 12.7% received new psychoactive drugs compared to only 5.0% of controls who were hospitalized in the same hospital during the same period. Within 3 months post-hospitalization, 0.5% of survivors received a new psychiatric diagnosis compared to 0.2% in the control groups (Wunsch et al. 2014).

#### Generalized anxiety disorder

While the lifetime prevalence of generalized anxiety disorder is 60% higher in females, with the highest prevalence amongst the 30–44 years age group, neither age nor sex were associated with anxiety symptoms after critical illness (Kessler et al. 2005). Severity of illness, length of hospital stay, and admission diagnosis were consistently found to have no association with anxiety symptoms that developed. Physical rehabilitation and diaries showed benefit among survivors of critical illness with anxiety (Spitzer et al. 1995).

#### Major depressive disorder

Major depressive disorders are twice as common in females (Kessler and Bromet 2013), with the highest prevalence amongst the 40–59 years age group (Pratt and Brody n.d.). Similar to generalized anxiety disorders, risk factors including age, sex, severity of illness, length of stay, and admission diagnosis were not associated with symptoms of major depression in survivors at follow-up (DiPietro 2001). For many patients, anxiety and depression are often comorbid with, or secondary to, post-traumatic stress disorder.

#### Post-traumatic stress disorder

Post-traumatic stress disorder after critical illness is associated with poor health-related quality of life, exacerbations of medical conditions, and cardio-respiratory, musculoskeletal, and gastrointestinal issues. Behaviors associated with post-traumatic stress disorder (i.e. avoidant coping, sleep disturbances, and substance use) may contribute to or worsen the above health issues (Pacella et al. 2013). The long-term psychological effects that follow sepsis, and their ability to be functionally debilitating, are important determinants of the morbidity and mortality of survivors.

### Physical impairment

#### The burden of survivorship

Physical disability is both a risk to one’s health and a major financial burden. Irreversible functional impairment plays a large role in decision making by patients that survive critical illness (Fried et al. 2002). Disability places an added burden upon the caregivers and families of the disabled (Langa et al. 2001) and is associated with increased rates of mortality (Rozzini et al. 2005). In addition, a decline in physical function is associated with a vast increase in cost of healthcare over the years that follow (Fried et al. 2001). The physical morbidity includes, but is not limited to, neuromuscular dysfunction (neuropathy, myopathy, and dysphagia), heterotrophic ossification, frozen joints, pulmonary dysfunction, tracheostomy issues, and compression neuropathy (Adhikari et al. 2010).

#### Development of new functional limitations

Iwashyna et al. (2010) assessed functional status in patients by comparing their functional status before and after sepsis. Functional status was determined by adding the number of the six activities of daily living (dressing, eating, ambulating, restroom use, getting in and out of bed, and daily hygiene) and five instrumental activities of daily living (preparing a hot meal, shopping for groceries, making telephone calls, taking medicines, and managing finances) requiring assistance to create a total deficiency score (from zero requiring no assistance to 11 requiring assistance for all categories). Patients with no limitations before sepsis developed a mean of 1.57 limitations. Patients with mild to moderate limitations before sepsis, developed a mean of 1.50 limitations. Hospitalizations for reasons other than sepsis were associated with the development of fewer new limitations. Worsening physical function was found to continue for at least 8 years, and post-sepsis functional disability was found to be independent of patient status before sepsis (Iwashyna et al. 2010). This landmark study found new, lasting limitations in physical activity among sepsis survivors with no or mild to moderate preexisting limitations.

While sepsis survivorship is a significant burden on the health of the individual patient, little consideration has been given to the effect of survivorship on the population at large. Sepsis stands in stark contrast to cancer and stroke survivorship, where survivorship is a known and crucial component of management and public health burden. Caregivers and medical professionals caring for survivors of cancer and stroke are aware of and vigilant for the physical and emotional effects of such a diagnosis. With sepsis, the population burden of survivorship is a newfound area for education and intervention. According to Medicare data, there are more than 500,000 survivors of sepsis with functional disability. Education for caregivers, programs targeting early mobility (Schweickert et al. 2009), delirium prevention (Girard et al. 2010), and continued physical and psychological services could be a major public health initiative and offer improved patient rehabilitation.

### Rehospitalization

#### Reasons for rehospitalization

Survivors of sepsis often find themselves back in the hospital. Studies show a 30-day rehospitalization rate between 20 and 32%, a 90-day rehospitalization rate of 40%, and a one-year rehospitalization rate of 63%. Causes for rehospitalization range widely, including systemic infection, redevelopment of sepsis, and exacerbation of previous chronic illness. Infection is the most common cause for rehospitalization with 12% of patients being re-admitted for infection post-sepsis (Prescott et al. 2015). The lungs are the most common sites of infection with pneumonia being the most common infectious process (Wang et al. 2014). Post-sepsis syndrome is marked by increased rates of infection along with exacerbation and development of chronic illness and organ dysfunction. This may be due to both impaired immunity and gut dysbiosis resulting from sepsis. In the acute phase, sepsis has both pro- and anti-inflammatory effects as a result of both innate and adaptive immune dysregulation (Shankar-Hari and Rubenfeld 2016). After cessation of these effects, survivors appear to be immunosuppressed with major defects in both innate and adaptive immunity; defective immunity leaves the host susceptible to infection (Hotchkiss et al. 2013).

#### Gut dysbiosis as a risk for infection

In terms of specific microbial effects, sepsis causes a dysbiosis in the gut that alters the microbiome from one rich in obligate and facultative anaerobes as found in the healthy gut, to one ripe with opportunity for pathogens to thrive (Pham and Lawley 2014). These changes were well established in several studies by Reeves et al. (2011) describing a murine model in which changes in the microbiota resulted in increased colonization by *C. difficile (*Reeves et al. 2011*)*. Overuse of antibiotics is especially problematic, with broad-spectrum antibiotics identified as a major cause of dysbiosis. Notably, clindamycin has been shown to trigger opportunistic infection by *C. difficile.*^*76*^ Buffie et al. (2012) showed that even a single dose of clindamycin causes significant change in the microbiota, with effects lasting for a minimum of 28 days and resulting in a loss of approximately 90% of normal microbial taxa from the cecum (Buffie et al. 2012). These findings shed light and provide mechanistic insights into infection as the most common cause for rehospitalization in sepsis survivors. Patients are often placed on multiple treatment regimens that include broad spectrum antibiotics as part of their disease management. It is possible that the immunosuppression that ensues as a result of the primary disease insult combined with microbial dysbiosis resulting from both the disease and treatment may be sufficient to cause new-onset sepsis (Iacob and Iacob 2019). Recent studies have shown that 6.4% of sepsis survivors aged 65 years and older were re-admitted within 90 days for new-onset sepsis (Prescott et al. 2015). Similarly, a Taiwanese study of 10,818 survivors of sepsis found a 35% risk of redeveloping sepsis (Shen et al. 2016).

The relationship between reinfection and post-sepsis syndrome is not limited to immunosuppression and dysbiosis, but also linked to cognitive and neuromuscular impairment that are further described in this review. For the purposes of this section, it is important to note that neuromuscular issues result in an increased risk of aspiration and, consequently, aspiration pneumonia. In a study of patients discharged from the intensive care unit, 63% of survivors of sepsis were found to have had aspiration compared to 23% of patients without sepsis (Zielske et al. 2014).

#### Development and exacerbation of medical conditions

Another reason for readmission of sepsis survivors is acute exacerbation of preexisting conditions. Patients with severe sepsis are generally older and sicker than the general population and typically harbor one or more chronic illnesses. Yende et al. (2014) found that 26% of sepsis survivors had chronic cardiovascular disease and 30% had a cardiovascular event within the past year. Similarly, 37% of these patients had diabetes, 31% had chronic lung disease (with 12.7% of patients experiencing acute exacerbation), and 10% had chronic kidney disease (Yende et al. 2014).

Several studies have found sepsis to be an independent risk factor for the development of disease without a prior diagnosis. In a study of patients aged 65 years and older who were readmitted within 90 days of discharge, 3.3% were readmitted for acute kidney injury, 5.5% were readmitted for congestive heart failure, and 1.9% were re-admitted for chronic obstructive pulmonary disease. Another study showed that patients discharged after sepsis had a 2.7-fold increased risk of acute kidney injury compared to matched controls (Prescott et al. 2015). Acute cardiovascular events occurred in 29.4% of survivors compared to 13.1% for unmatched controls (Yende et al. 2014). The effects that sepsis has on the cardiovascular system are notable in particular and will be elaborated upon below. Sepsis and its elevated inflammatory markers have been shown to accelerate atherosclerosis via endothelial injury (Kaynar et al. 2014). Persistent vascular inflammation can cause disruption of stable plaques and lead to cardiovascular events.

#### Cardiovascular disease after sepsis

Infection is a major trigger for acute coronary syndromes by producing demand ischemia, endothelial dysfunction, procoagulant states, and inflammatory cellular infiltration (i.e. T-cells, macrophages, and neutrophils) within atherosclerotic plaques. These infectious sequelae can increase the short-term risk of cardiovascular events including stroke, myocardial infarction, and fatal coronary artery disease (Corrales-Medina et al. 2010; Corrales-Medina et al. 2013). The recruitment of inflammatory cells contributes to acute coronary syndromes by producing cytokines, proteases, coagulation factors, free radicals, and vasoactive intermediates that increase endothelial damage, disrupt the fibrous cap, and start the formation of thrombi (Hansson et al. 2006). In addition, the residual inflammatory and procoagulant state can extend for years after the infection resolves and raise a survivor’s risk of cardiovascular diseases (Yende et al. 2008; Yende et al. 2011). Hospitalization for severe pneumonia carried a four-fold increase in developing cardiovascular disease in the first 30 days post-infection and remained elevated for 10 years (Corrales-Medina et al. 2015).

Both atherosclerosis and sepsis are inflammatory states that are associated with increased short- and long-term risk of cardiovascular disease. Yende et al. (2014) found that survivors of severe sepsis had a 13-fold increased risk of developing cardiovascular events compared with unmatched controls and a 1.9-fold increased risk compared with matched-population controls (Yende et al. 2014). This 1.9-fold increase in risk is similar to the risk of developing cardiovascular events in cigarette smokers and patients with diabetes and hypercholesterolemia (Wilson et al. 1998). In contrast to the other organ systems, the cardiovascular system is unique in that 45% of survivors did not have preexisting cardiovascular disease before the hospitalization for sepsis, yet 25.9% of these patients developed subsequent cardiovascular events (Yende et al. 2014). These findings illustrate that post-sepsis syndrome may predispose survivors to the development of new diseases or chronic illnesses where previously none existed.

### Clinical considerations and paths forward

#### Therapeutic strategies

Despite a vast increase in sepsis survivorship over the last two decades, there are no widely established guidelines for post-sepsis syndrome. Current protocols aim to reduce short-term mortality, but little consideration has been given to the prevention of cognitive, psychological, medical, and functional consequences. Prescott et al. (2018) offered some key strategies in preventing long-term complications after sepsis: effective care during early sepsis; controlling pain, agitation, and delirium; and early mobilization. Early hospital care for sepsis emphasizes rapid detection, administration of broad-spectrum antibiotics, removing sources of infection (i.e. infected indwelling catheters), and resuscitation with intravenous fluids and vasopressors for patients with hypotension or elevated lactate (Rhodes et al. 2017). Pain, agitation, and delirium are common with sepsis and are associated with an increased risk of mortality, cognitive impairment, and post-traumatic stress disorder (Reade and Finfer 2014). Promoting early and progressive activity (bed-based exercises, to sitting, standing, and ultimately walking), was found to result in speedier time to physical therapy and ambulation, as well as shorter duration of delirium during hospitalization (Schweickert et al. 2009). Several authorities suggest that rehabilitation with physical, occupational, and speech therapy benefits survivors of sepsis who develop new weakness (Connolly et al. 2016; Major et al. 2016). Clinicians should assess patients for common and preventable causes of hospital readmission (infection, congestive heart failure exacerbation, acute kidney injury, chronic obstructive pulmonary disease exacerbation, and aspiration pneumonia) and modify delivery of care to anticipate and prevent these complications (Table 2) (Prescott and Angus 2018).

**Table 2:** Key strategies in preventing long-term complications after sepsis

Key strategies in preventing long-term complications after sepsis	
1. Early sepsis care Elements of care: antibiotics, fluid resuscitation, vasopressors, control source of infection Guideline: Surviving Sepsis Campaign: international guidelines for the management of sepsis and septic shock (SSC) (Rhodes et al. 2017)	
2. Pain, agitation, and delirium management Elements of care: pain assessment, pain treatment, sedative choice, sedative monitoring, depth of sedation, delirium monitoring Guideline: clinical practice guidelines for the management of pain, agitation, and delirium in adult patients in the intensive care unit (PAD) (Barr et al. 2013)	
3. Early mobility Elements of care: mobilization Guideline: National Institute for Health and Care Excellence (United Kingdom): clinical guideline on rehabilitation after critical illness (NICE) (Cotton 2013)	

#### Rising incidence of sepsis

In order to properly contextualize the newfound importance of post-sepsis syndrome, it is necessary to examine both the rising incidence of sepsis and the decrease in mortality from sepsis. There are two likely explanations for the rising number of cases over the last few decades: larger populations at risk and improved recognition. A growing elderly population that is at higher risk of sepsis, as aging is a risk factor (Martin et al. 2006). Immunosuppressive regimens have become more common for patients with cancer, rheumatologic conditions, and solid-organ transplantation. These patients are at a higher risk for acquiring infections. Second, the increase in estimates may be due to improved methodology related to current epidemiologic studies. Sepsis became a focal point in critical care and medical literature with recognition that early treatment leads to better outcomes. Earlier recognition is perhaps the reason for both reported increases in sepsis incidence and lower case-fatality rates due to timely diagnosis of more-manageable cases.

#### Decrease in mortality from sepsis

The decrease in case-fatality rates from sepsis are likely due to enhanced critical care support, standardized protocols supported by international guidelines, and, as above, early recognition. With the case-fatality rate down, we must still define, anticipate, and treat the sequelae of sepsis. While the long-term sequelae of sepsis have been loosely grouped together until this point, there is an apparent need for better understanding of the underlying pathophysiological mechanisms and organizing these sequelae to guide treatment.

Consideration of post-sepsis syndrome as a discrete syndrome is only two decades old; nonetheless, its prevention has become an important topic of current exploration. The first and foremost preventive measure is optimization of sepsis management in order to prevent injury to the vital organs; the Surviving Sepsis campaign guidelines are the mainstay of optimization (Dellinger et al. 2013). Patients with a chronic disease should be closely monitored for exacerbations of illness and worsening organ function as has been shown to occur in post-septic patients. Many patients are not known to have cardiovascular disease before sepsis and would benefit from primary prevention interventions during their hospitalization (e.g. statin or aspirin). Intensive Care Experience Questionnaire (ICEQ) combined with discharge assessment of emotional state may provide a simple way of predicting which patients may develop psychiatric sequelae (Rattray et al. 2004). Cognitive impairment has been the most closely examined effect of post-sepsis syndrome as it is also the most worrisome for clinicians, families, and patients. Appropriate control of blood glucose, especially hypoglycemic events and rapid changes in blood glucose, may reduce the risk of subsequent cognitive impairment (Annane and Sharshar 2015). Some proposed interventions may restore the blood-brain barrier, reduce cortical oxidative stress, and decrease microglial activation. These treatments could be the subjects of future sepsis trials; these include hydrocortisone, minocycline, erythropoietin, haemin, immunoglobulins, and recombinant C5a (Annane and Sharshar 2015).

#### Paths forward

**Fig. 5:**
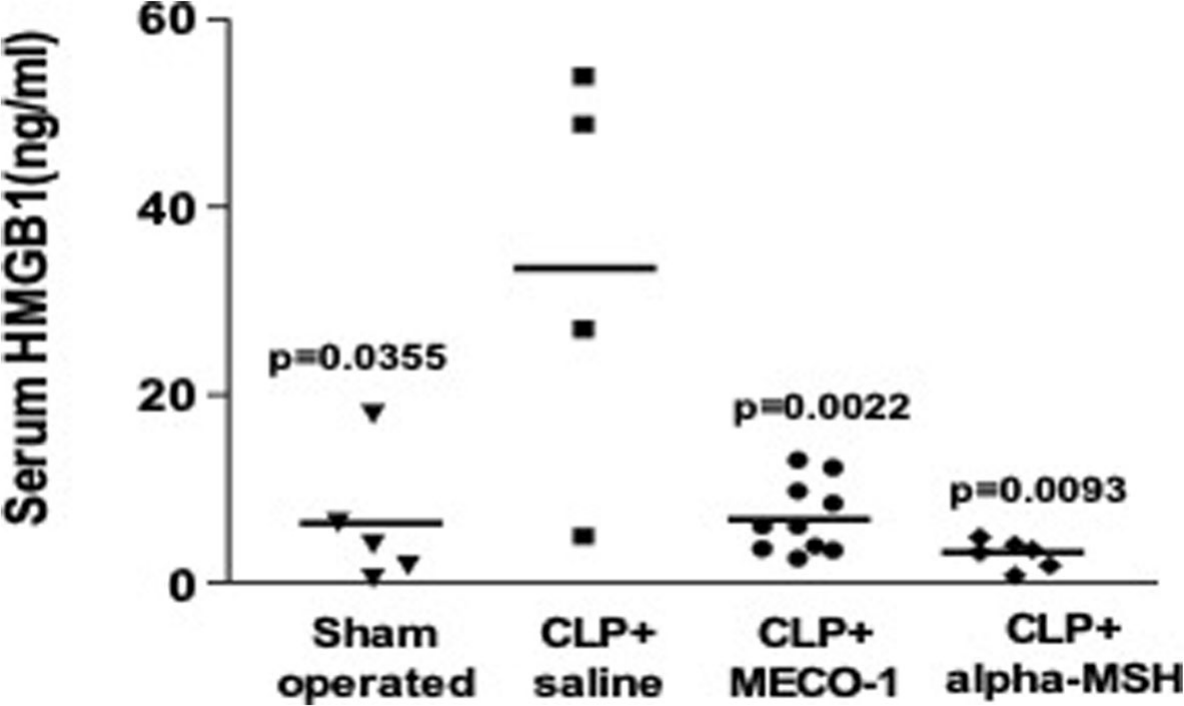
MECO-1 and α-MSH as anti-HMGB1 corticotropic peptides. Figure used with permission from authors (Qiang et al. 2017). Melanocortin-like peptide of *E. coli*-1 (MECO-1) has anti-inflammatory effects similar to α-melanocyte-stimulating hormone (α-MSH) and adrenocorticotropin (ACTH), two prominent mammalian melanocortin hormones. HMGB1, the proposed major inflammatory mediator of the post-sepsis syndrome, remained elevated in septic mice treated with saline alone. MECO-1 and α-MSH were equally effective in attenuating the release of HMGB1 from macrophage-like cells in septic mice. The model for sepsis used was cecal ligation and puncture (CLP)

While targeted treatments for post-sepsis syndrome are not currently available, several potential interventions are under investigation. One promising therapy is targeting HMGB1, the presumed major mediator of post-sepsis syndrome and the accompanying cognitive decline. The microbiota has been linked to nearly every arm of the immunogenic cascade. These native bacteria are directly responsible for suppressing infectious invaders and initiating a measured immune response. Many microbial peptides including melanocortin-like peptide of *E. coli*-1 (MECO-1), a peptide synthesized by *E. coli,* have been found to suppress the release of HMGB1 by macrophages. MECO-1 is at least as effective as major endogenous mammalian corticotropins, namely, alpha-melanocyte stimulating hormone (alpha-MSH) and adrenocorticotropin (ACTH) (Qiang et al. 2017). Melanocortins produced by commensal organisms may prevent or hinder development of post-sepsis syndrome by inhibiting the inflammatory mediators of overwhelming inflammation that follows sepsis (Fig. 5).

## Conclusions

Improved outcomes for patients with sepsis, combined with a rise in survivorship, have warranted considerable research and clinical efforts focusing on life during and after sepsis. Recent studies in both mice and humans have found that survivors of sepsis are subject to several categories of complications. The categories of long-term effects of sepsis include immunometabolic, neurocognitive, psychiatric, and physical derangements. These effects lead to increased mortality and severely worsened health-related quality of life. Health care professionals and family members pay a heavy cost—in both money and energy—in caring for these patients with sizable health issues. This period, known as post-sepsis syndrome, is an entity of newfound importance and the subject of much exploration. The current goals of care for patients who survive sepsis include approaches that are effective in early sepsis, controlling pain, agitation, and delirium, and early mobilization. However, there is an unmet need of developing efficient therapeutic strategies specifically tailored towards sepsis survivors. Preclinical research has identified promising new therapeutic targets for preventing and treating the post-sepsis syndrome. The validity of therapeutic approaches successfully indicated in ongoing preclinical research remains to be established in clinical settings.

## Data Availability

Not applicable.

## Abbreviations

HMGB1: High mobility group box-1
MECO-1: Melanocortin-like peptide of *E. coli*-1
